# Evaluation of an educational concept for risk-oriented prevention in undergraduate dental education

**DOI:** 10.1186/s12909-020-02218-x

**Published:** 2020-09-11

**Authors:** Gerhard Schmalz, Felix Krause, Martin Grzelkowski, Cordula Merle, Daisy Rotzoll, Rainer Haak, Dirk Ziebolz

**Affiliations:** 1 Department of Cariology, Endodontology and Periodontology, University of Leipzig, Liebigstr. 12, 04103 Leipzig, Germany; 2 Department of Operative Dentistry, Periodontology and Preventive Dentistry, RWTH Aachen University, Aachen, Germany; 3 Department of General Medicine, Faculty of Medicine, University of Leipzig, Leipzig, Germany; 4 University of Leipzig, Medical Faculty, LernKlinik Leipzig, Leipzig, Germany

**Keywords:** Dental education, Risk classification, Prevention

## Abstract

**Background:**

Aim of this observational study with a three-month follow-up was to evaluate an educational concept for risk-oriented prevention applied by fifth-year undergraduate dental students.

**Methods:**

Dental students from two clinical treatment courses of the last undergraduate year were included. The subjects were divided into two groups according to their assignment to the two clinical classes. Group A received a sequence of seminars, including the basics of a risk classification system (RCS) with the theoretical background and case studies in the context of preventive dentistry. Thereby, 1) a theoretical seminar (background, RCS, cases) and 2) the transfer of the RCS on a clinical patient case chosen by the student, and its presentation within a discussion round was applied. Group B served as a comparison group with students who did not receive any of teaching events in terms of RCS. The self-perceived knowledge and importance of RCS, as well as objective knowledge (qualitative questions), were assessed with a standardized questionnaire at baseline and after 3 months.

**Results:**

Out of 90 students at baseline, 79 (group A: 39, group B: 40) were re-evaluated after 3 months. At this follow-up, Group A estimated their confidence in handling the medication (*p* = 0.02), the RCS (*p* < 0.01), and in identifying the risk of oral diseases (*p* = 0.02) higher than group B. Furthermore, group A felt it was more important to identify patients at risk (*p* = 0.02), the risk of complications (*p* = 0.02) and to apply an RCS (*p* = 0.03). At follow-up, group A exhibited more correct answers of qualitative questions than group B regarding risk of complications (*p* < 0.01) and bacteremia (*p* < 0.01). Group A felt more confident with at-risk patients and more competent concerning RCS than group B (*p* < 0.01).

**Conclusion:**

The concept for educating risk-oriented prevention increased the self-perceived skills and the knowledge of undergraduate dental students after 3 months within a clinical treatment course.

## Background

Related to the aging of the world’s population, the prevalence of chronic diseases increased during the past decades [1]. This development is of high relevance for dental care for two main reasons. On the one hand, patients with chronic diseases or conditions and possibly with concomitant medication can suffer from a risk of complications due to dental interventions, e.g., infective endocarditis or systemic infections following invasive measures [2, 3]. On the other hand, several systemic parameters are related to the oral cavity and can increase the risk of development, progression, and severity of oral diseases, e.g., periodontitis [4, 5]. Thereby, bidirectional relationships are conceivable for several constellations, e.g. periodontal disease and diabetes mellitus [6] or rheumatoid arthritis [7, 8]. Especially in dental prevention, a shift from a surgical to a medical focus is, therefore, recommendable [9]. Accordingly, sufficient knowledge of dentists with regard to systemic diseases and the measures for prevention of complications (e.g. antibiotic prophylaxis) seem mandatory; in contrast, dentists knowledge about these topics and their interdisciplinary collaboration with general physicians is limited [10–14].

Against this background, it is of increasing relevance for undergraduate dental students to be prepared for future challenges in the dental care of patients with general diseases, conditions and/or medications [15]. Thereby, awareness and knowledge of antimicrobial prophylaxis need to be strongly emphasized [16]. Similarly, oral and systemic disease interrelation should be included in dental curricula as an important part [17]. Additionally, interprofessional training of dental and general medical students should be established to promote interdisciplinary collaboration at an early stage [18]. For these issues, different approaches have been discussed. It was shown that a one-day course can improve dental students’ knowledge towards anticoagulation [19]. Another study effectively used standardized patients for teaching interprofessional issues in dental and medical students [20]. For patients with dental special care needs, the students’ own clinical experience was an effective learning approach, which led to increased self-efficacy and sensibilization for the topic [21]. These approaches are only related to each one sub-aspect of the students´ management of at-risk patients. A comprehensive concept, which helps the students to evaluate and classify different types of risk patients with both a risk of complications and a risk of oral diseases, has not been evaluated, yet.

This observational study with a three-month follow-up aimed to evaluate a teaching concept for risk-oriented prevention in fifth-year undergraduate dental students within the clinical treatment courses. Thereby, a risk classification system (RCS) was imparted within two seminars, including a presentation of students’ patient cases during their clinical course. The RCS supports the assessment of a risk of complications and a risk of oral diseases of patients in dental prevention. Thereby, one out of three risk classes (low, moderate or high) for each respective general disease, medication or lifestyle parameter is defined according to the risk profile of individualized prevention (supplementary Table 1) [22]. It was hypothesized that the transfer of an RCS in the students’ patient treatment could increase their self-perceived skills and the importance of risk patients as well as students’ knowledge in risk classification.

## Methods

### Study design

This current study was designed as an observational teaching study with a three-month follow-up. The study was approved by the Ethics Committee of the medical faculty of Leipzig University, Germany (No. 378/15–05102015). All participating students were informed verbally and in writing and gave written informed consent.

### Participants and groups

All participants were dental students within a clinical patient course in the fifth year of undergraduate studies. During the investigation, half of the students received a seminar about risk-oriented prevention. The group allocation was defined by the assignment to the particular clinical course at baseline. This resulted from the course of the students’ studies and was made independently of this investigation. Accordingly, there was no randomization or matching of the two groups. Group A underwent the course in cariology, endodontology, and periodontology and thereby received two seminars on risk-oriented prevention. Group B participated in the course of prosthodontics and did not receive any lessons on risk-oriented prevention.

### Teaching of the risk classification system

**Fig. 1:**
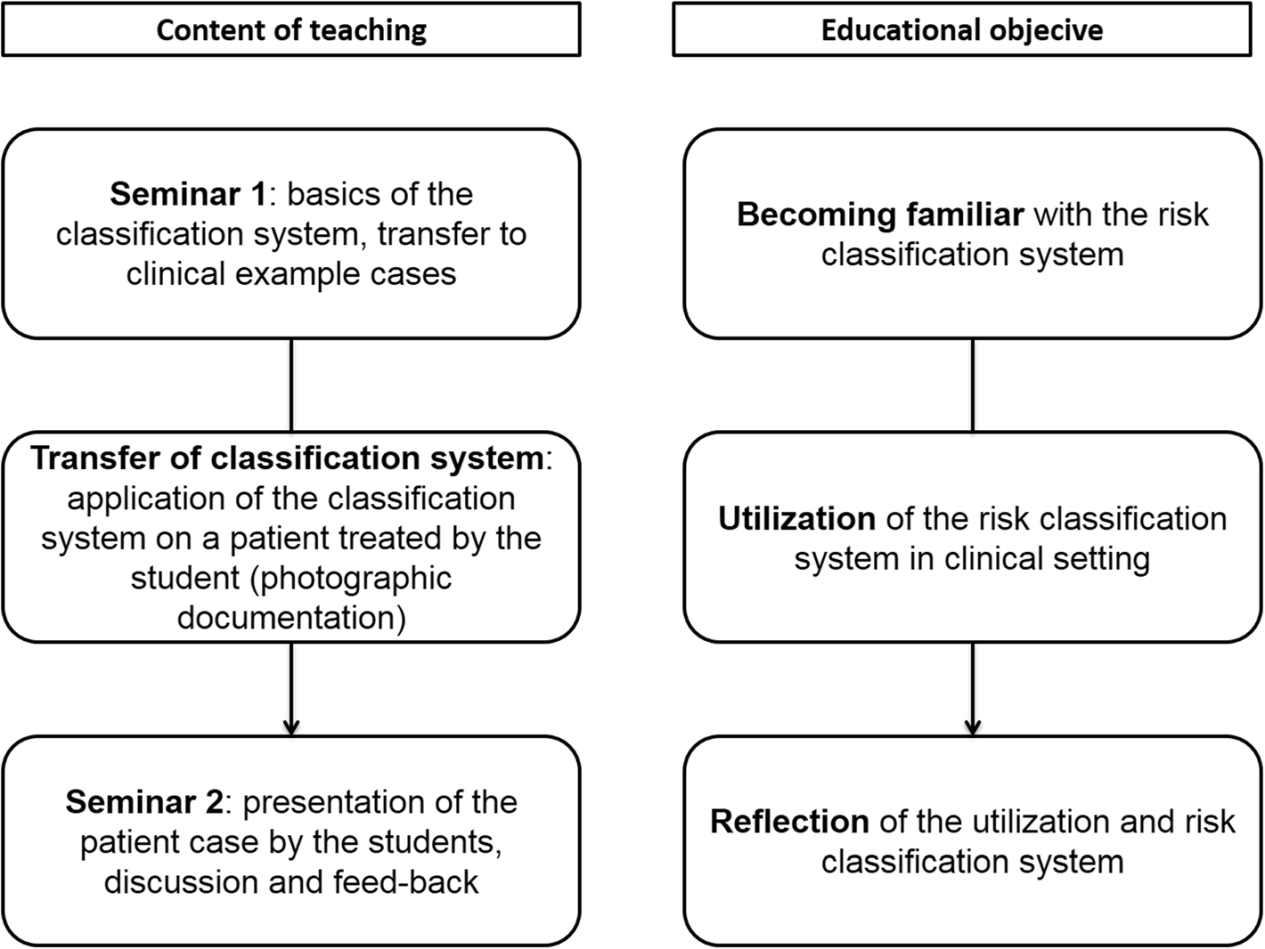
Educational concept for risk-oriented prevention, including teaching events and objectives

All students included in group A received two structured seminars regarding risk-oriented prevention. The educational concept consisted of two core elements: 1) the first seminar with basics of the RCS and its implications for dental preventive measures, as well as 2) the transfer of the RCS on student’s patient cases and the presentation of the results in a second seminar (discussion round). In the first seminar, the RCS was explained and applied on example patient cases with the students in small-group work (groups of 3–4 students, randomly composed by drawing of lots). The basis of risk-oriented prevention was the concept of risk classification, as already described by this working group [22]. In brief, two risks are defined for each patient: risk of complications, and risk of oral diseases based on the underlying disease, medication, or lifestyle factor. Each risk factor can be assigned a risk class out of low (e.g. generally healthy), moderate (e.g. well-controlled diabetes mellitus leading to a moderately increased periodontitis risk), or high (e.g. heart valve replacement leading to the necessity of antibiotic prophylaxis) (supplementary Table 1). During the semester flow, students chose a patient case by themselves and utilized the classification system for risk assessment. In the second seminar, the students presented the results of their risk classification to each other in a roundtable group discussion (Fig. 1).

### Questionnaires

All students answered a questionnaire both at the beginning and at the end of the clinical training course (baseline and after 3 months). This questionnaire included different issues about the applied RCS. The assessment included sociodemographic information (age, gender, medical pre-education), self-perceived knowledge/skills, and self-perceived importance of several issues within risk-oriented prevention. Several qualitative questions were composed to assess an objective gain in knowledge. For ten patient cases each, students had to classify whether these patients have a low, moderate, or high risk of complications and/or of oral diseases, respectively. Furthermore, it was asked whether these ten cases would need antibiotic prophylaxis. Five questions were formulated, addressing the risk and magnitude of bacteremia related to dental measures. In addition, several questions regarding the interdisciplinary view of the dental students were asked. Finally, a short self-reflection was included, which evaluated whether students feel confident in treating at-risk patients, and if they experienced a benefit from the seminars (only group A).

### Study flow

At the beginning of the semester and before the seminars, all students who provided informed consent answered the questionnaire. While group A received the two seminars as described above, group B did not undergo any teaching session in risk-oriented prevention. After group A had completed the whole seminars, all participants answered the questionnaire again. Two semesters (i.e. summer term 2019 and winter term 2019/20) were used to include respective students for this study to achieve a preferably high sample size. The questionnaire-based evaluation was performed anonymously.

### Statistical analysis

The statistical analysis has been performed with SPSS for Windows, version 24.0 (SPSS Inc., U.S.A.). The results of qualitative questions were summarized as the percentage of correct answers (e.g. 7/10 correct answers means 70% correct). Normal distribution was tested with Kolmogorov-Smirnov-test, whereby no normal distribution was found. Accordingly, Mann-Whitney-U test was applied as a non-parametric test. Categorical data were analyzed using chi-square or fisher-test, respectively. The significance level was set at *p* < 0.05.

## Results

### Participants

At baseline, 90 of fifth-year dental students were included in the investigation, with 45 participants each group (A and B). Of these students, 79 (group A: 39, group B: 40) were re-evaluated after 3 months, because 11 students lost their follow-up. The age (26.02 ± 3.70 vs. 24.71 ± 3.15 years, *p* = 0.08) and gender distribution (38% vs. 24% male participants, *p* = 0.26), as well as the presence of medical pre-education (27% vs. 9%, *p* = 0.06), was not significantly different between group A and B.

### Subjectively experienced issues regarding risk classification and identification of at-risk patients

At baseline, both groups evaluated their skills in and the importance of risk classification similarly. Thereby, both A and B stated a high importance of identification of at-risk patients in dental prevention (4.40 ± 1.32 vs. 4.22 ± 1.51, *p* = 0.72; Table 1). At the follow-up evaluation, group A rated higher confidence with medication (3.28 ± 0.56 vs. 2.97 ± 0.58, *p* = 0.02), better skills in risk classification (3.33 ± 0.77 vs. 2.92 ± 0.57, *p* < 0.01) and better identification of risk of oral diseases (3.74 ± 0.55 vs. 3.35 ± 0.77, *p* = 0.02) than group B. Furthermore, group A rated the identification of at-risk patients (4.72 ± 0.92 vs. 4.33 ± 1.12, *p* = 0.02), the risk classification (4.23 ± 0.96 vs. 3.78 ± 1.10; *p* = 0.03) and the identification of complication risk (4.69 ± 0.92 vs. 4.30 ± 1.11, *p* = 0.02; Table 1) more important.

**Table 1:** Subjectively experienced issues regarding risk classification and identification of at-risk patients in dental prevention at baseline and follow-up (after 3 months), values are given as mean values ± standard deviation; 1 = not at all, 5 = very good/very important. Significant values (*p* < 0.05) are highlighted in bold

	Baseline			Follow-up		
	Group A (***n*** = 45)	Group B (***n*** = 45)	***p***-value	Group A (***n*** = 39)	Group B (***n*** = 40)	***p***-value
**How confident are you with general diseases?**	3.11 ± 0.71	2.91 ± 0.85	0.17	3.56 ± 0.60	3.37 ± 0.59	0.12
**How confident are you with medication?**	2.91 ± 0.73	2.89 ± 0.86	0.73	3.28 ± 0.56	2.97 ± 0.58	**0.02**
**How confident are you with general diseases + medication?**	2.84 ± 0.74	2.82 ± 0.96	0.73	3.10 ± 0.72	2.88 ± 0.65	0.10
**How good is your identification of at-risk patients?**	3.33 ± 0.74	3.20 ± 0.84	0.50	3.85 ± 0.81	3.55 ± 0.78	0.18
**How good do you master risk classification?**	2.78 ± 0.82	3.00 ± 0.77	0.14	3.33 ± 0.77	2.92 ± 0.57	**< 0.01**
**How good do you identify a risk of complications?**	3.16 ± 0.77	3.00 ± 0.80	0.28	3.46 ± 0.55	3.23 ± 0.62	0.09
**How good do you identify a risk of oral diseases?**	3.07 ± 0.78	2.91 ± 0.85	0.53	3.74 ± 0.55	3.35 ± 0.77	**0.02**
**How important is the identification of at-risk patients for you?**	4.40 ± 1.32	4.22 ± 1.51	0.72	4.72 ± 0.92	4.33 ± 1.12	**0.02**
**How important is risk classification for you?**	3.87 ± 1.24	3.91 ± 1.20	0.92	4.23 ± 0.96	3.78 ± 1.10	**0.03**
**How important is identification of risk of complications for you?**	4.33 ± 1.37	4.13 ± 1.47	0.39	4.69 ± 0.92	4.30 ± 1.11	**0.02**
**How important is identification of risk of oral diseases for you?**	4.18 ± 1.35	4.07 ± 1.40	0.68	4.54 ± 0.85	4.20 ± 1.02	0.08

### Qualitative assessment of students’ knowledge

At the baseline evaluation, the knowledge of the participants to correctly determine the risk of complications, risk of oral diseases, accurate indication of antibiotic prophylaxis, and exact magnitude of bacteremia due to dental interventions was similar in both groups (*p* > 0.05). At follow-up, group A exhibited higher skills than group B, which was significant for risk of complication (58.97 ± 13.53 vs. 44.75 ± 21.84, *p* < 0.01) and bacteremia (86.67 ± 14.02 vs. 75.00 ± 15.53, *p* < 0.01; Table 2).

**Table 2:** percentage distribution of amount of correct answers for the four qualitative question complexes, including correct determination of risk of complications, risk of oral diseases, correct indication of antibiotic prophylaxis and correct magnitude of bacteremia due to dental interventions in %. Significant values (*p* < 0.05) are highlighted in bold

	Baseline			Follow-up		
	Group A (***n*** = 45)	Group B (***n*** = 45)	***p***-value	Group A (***n*** = 39)	Group B (***n*** = 40)	***p***-value
**Risk of complications**	51.78 ± 19.10	56.00 ± 14.52	0.27	58.97 ± 13.53	44.75 ± 21.84	**< 0.01**
**Risk of oral diseases**	45.11 ± 19.50	42.89 ± 18.17	0.48	53.85 ± 15.83	44.50 ± 21.95	0.09
**Indication of antibiotic prophylaxis**	78.67 ± 7.26	78.67 ± 7.86	0.91	80.00 ± 7.95	76.75 ± 13.47	0.30
**Bacteremia**	82.22 ± 14.91	76.89 ± 16.49	0.11	86.67 ± 14.02	75.00 ± 15.53	**< 0.01**

### Evaluation of interdisciplinary view

The majority of participants at both time points stated that they would be willing to cooperate with a general physician after graduation (Table 3). At follow-up, the majority of group A rated the dentist to be responsible for the indication of antibiotic prophylaxis (89.7% vs. 61.5%, *p* = 0.01). In the opposite, group B rated the primary responsibility for this at the general physician (43.6% vs. 82.1%, *p* < 0.01; Table 3).

**Table 3:** Questions regarding antibiotic prophylaxis and interdisciplinary collaboration at baseline and follow-up given as percentage. Significant values (*p* < 0.05) are highlighted in bold

	Baseline			Follow-up		
	Group A (***n*** = 45)	Group B (***n*** = 45)	***p***-value	Group A (***n*** = 39)	Group B (***n*** = 40)	***p***-value
**Collaboration with general physicians after graduation**	90.7%	75.6%	0.09	94.7%	84.6%	0.26
**Indication of antibiotic prophylaxis by general physician**	68.2%	82.2%	0.23	43.6%	82.1%	**< 0.01**
**Indication of antibiotic prophylaxis by dentist**	79.5%	84.4%	0.59	89.7%	61.5%	**0.01**
**Indication of antibiotic prophylaxis according to guidelines**	77.3%	73.3%	0.81	92.3%	92.3%	0.99

### Self-reflection of students´ perspective

**Fig. 2:**
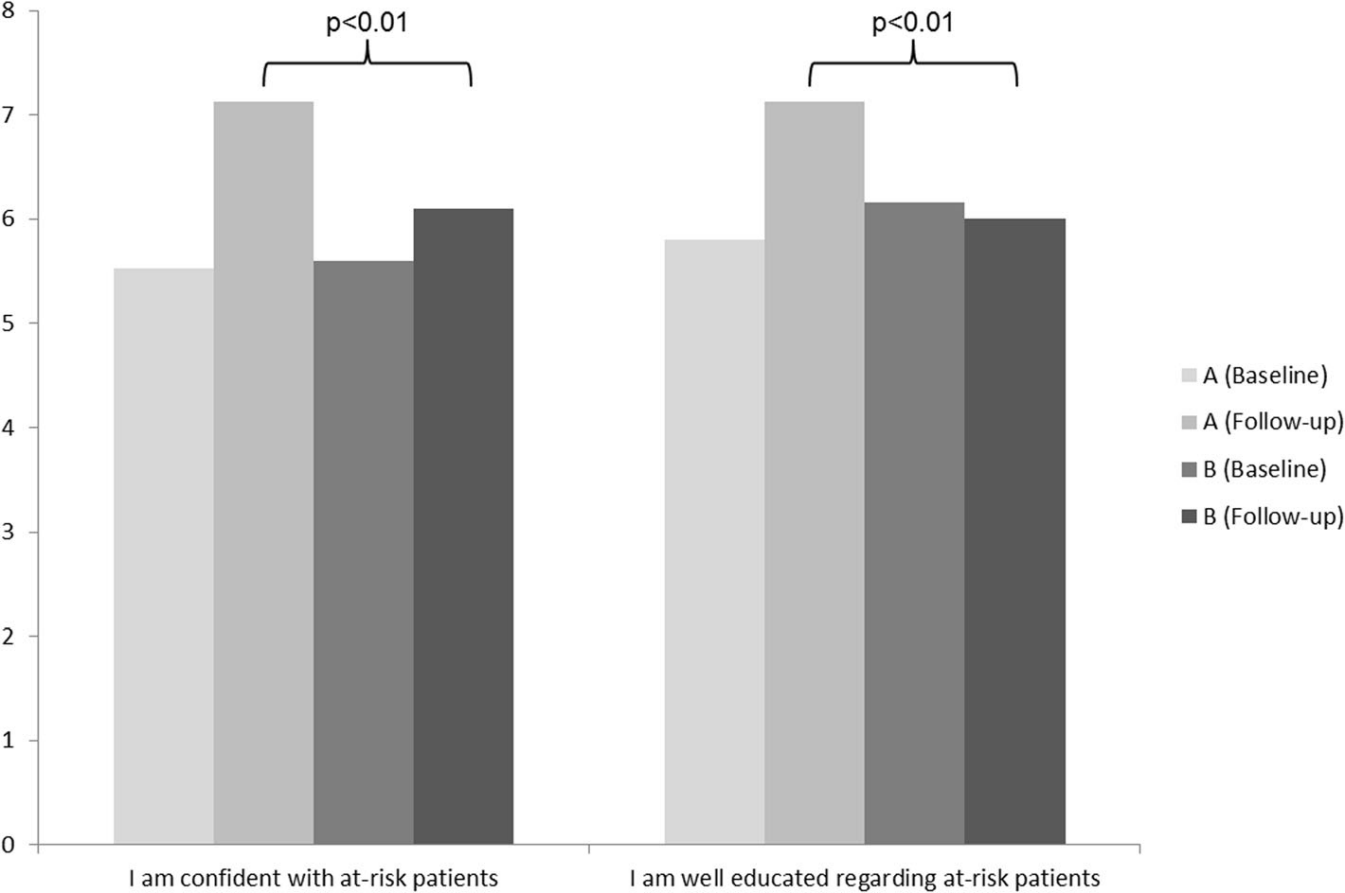
Student’s self-reflection regarding their perceived confidence with at-risk patients and education level regarding at-risk patients (1 = not correct at all, 10 = applies completely)

At the follow-up evaluation, group A felt more confident with at-risk patients than group B (7.13 ± 0.98 vs. 6.10 ± 1.71, *p* < 0.01). Participants of group A felt better trained with regard to RCS compared to group B (7.13 ± 1.15 vs. 6.00 ± 1.50, *p* < 0.01; Fig. 2). Group A perceived the average benefit of the seminar “risk-oriented prevention” including the RCS as high, with on average 8.87 ± 1.20 (1 = no benefit, 10 = highest benefit).

## Discussion

### Main results

Students who had received the two seminars about RCS felt more confident with risk classification and rated its importance higher than the comparison group. At follow-up, the students who underwent teaching had significantly better knowledge in determining the risk of complications caused by dental preventive measures and bacteremia related to dental interventions. Furthermore, the students who had received the seminars rated the responsibility for the indication of antibiotic prophylaxis mainly by the dentist. In contrast, the comparison group had seen this responsibility by the general physicians. At follow-up, specially trained students (group A) felt more confident and well-educated with at-risk patients than the control group B.

### Comparison with available literature

A previous German study found that undergraduate dental students treat a high number of patients with systemic internal diseases during their clinical semesters [15]. Thereby, it was also concluded that more studies focusing on medical topics in dental education would be necessary [15]. The current research has specifically trained students in their fifth year of dental education in a clinical treatment course with regard to an RCS for systemic diseases, medication, and lifestyle factors. The medical contents of this program have previously been described by this working group [22]. As the main teaching and learning approach, students transferred this RCS to a patient case, which they had chosen by themselves and presented their results within a group discussion with their colleagues. It is known that clinical experience and the reflection of own patient cases can increase both students’ interest and understanding of the particular issue [21, 23, 24]. This is a kind of case-based learning approach, which has been described to be superior in increasing the ability of students to make accurate clinical decisions compared with lecture-based teaching [25]. Accordingly, dental students were reported to prefer patient-case-based against lecture-based learning [26]. Additionally, the discussion of the patient case with other students might have a particularly positive effect [27]. These issues are in line with the results of the current study, where better perceived and objective knowledge was present in students who had received the additional education program. In addition, the transfer of one’s own knowledge to real clinical patient cases fulfils the requirement to link the teaching of theoretical contexts with actual practice in a clinical treatment setting [28].

Altogether, the approach of teaching RCS in the way as it was performed in the current study appears reasonable and advantageous for both students self-perceived and their objectively assessed knowledge in this field. However, the variety of general diseases, medications, and lifestyle factors are limited by the cases, which occur in the clinical course and by the students’ choice of patients [15, 29]. Therefore, the students’ patient cases might be complemented by sample cases chosen by the teacher to ensure a proper variety of different situations. Methodologically, this could be carried out as a standardized patient exercise, which has been shown to foster the knowledge and skills of students [20]. A particular benefit could be achieved by an interprofessional approach [18, 29]. This could provide a basis for future collaboration between dentists and general physicians and should, in the long term, improve the current situation, where an insufficient interdisciplinary collaboration is reported [12].

Because this is the first study evaluating a teaching concept for risk-oriented prevention including an RCS in fifth-year undergraduate dental students of a clinical education course, no directly comparable studies are available. One previous study conducted a one-day course in dental students regarding anticoagulation and dental treatment, whereby a considerable improvement was achieved by the students [19]. Other educational strategies were not reported, yet. Especially regarding safety in the dental care of medically compromised patients, a high need for education is seen [30]. Thereby, improved training strategies of dental students in medical issues are demanded [31]. The approach of the current study might be part of a respective curriculum, as it shows that students self-perceived and objectively assessed skills were improved (group A) compared to comparison group B. Although the seminars lead to an improvement, this effect was quite small, whereby still more than 40% of questions regarding the risk of complications were answered wrong in both groups. This might indicate a still high need for training in the field of risk-oriented prevention. The knowledge of participants regarding antibiotic prophylaxis was approximately good (over 75% correct answers), regardless of the methodological approach. In the available literature, mixed results were found for students’ knowledge regarding this issue [16, 32]. However, the students, who took part in the seminars (group A), stated that the dentist must make the indication for antibiotic prophylaxis. It may indicate that these students perceive a higher sense of responsibility for this topic. On the other hand, they still answered 40% of the questions regarding risk of complications and 20% of questions regarding antibiotic prophylaxis wrong. Therefore, the finding might also indicate that the RCS would give these students a false sense of confidence. Accordingly, the findings must be interpreted with caution. Furthermore, at follow-up, more than 90% of participants in both groups were able to prescribe antibiotics prophylaxis according to the guideline, without differences between groups. As this is the most clinically relevant issue, this finding appears to be most important in this context, and seem to be unaffected by the respective teaching events.

The students of group A rated the benefits of the two seminars for their RCS skills as high. They stated to feel better educated and more confident with risk-patients than the comparison group B. This is generally an expectable result, because group B had not received any seminars regarding RCS. However, it is also an argument for teaching the RCS, because it positively influenced the students view on the topic. It is known that a continuous self-reflection, e.g. via logbook can further increase the knowledge and motivation of students by reflecting their strengths and weaknesses regarding their clinical competence [33, 34]. However, the appropriate form and content of self-reflection can be discussed controversely [35]. Therefore, self-reflection in contrext of learning risk-classification might be a relevant issue that should be considered in the further implementation of risk-oriented prevention in curricular dental education. Moreover, the benefit of the education concept might be increased by an earlier implementation; a previous study regarding clinical reasoning competencies concluded that case-based learning should start as early as possible [25]. Therefore, the presented concept within this current study might be longitudinally included in the dental and medical education curriculum to increase its value.

### Strengths and limitations

This is the first study evaluating an education concept of risk-oriented prevention including a specific RCS in undergraduate fifth-year dental students. The sample size seems appropriate, with approximately 40 students in each group at the follow-up. However, the loss of 11 students during follow-up is a potential bias, because particularly primarily motivated students might have attended the follow-up appointment. Moreover, the statistical power remains unclear, whereby the sample size is limited by the number of students in each year and the voluntary participation. The groups were not composed during a randomization process, but were based on the separation of the students into two groups, which is a regular process independently of the current study. However, this can be seen as limitation. Thereby it must be mentioned that group A and B were studying different courses - group A was studying cariology, endodontology and periodontology, which has more emphasis on antibiotics prophylaxis than prosthodontics, which is generally more about biomaterials/biomechanics. This must be seen as a potential source of bias; splitting students within the same course would have been more appropriate, but would have lead to a lower sample size and to the exclusion of students from the curricular seminars regarding risk-oriented prevention. Because of the anonymous character of the investigation, no related samples could be analyzed, making the statistical analysis of an effect over the 3 months follow-up impossible. Therefore, the available analysis just allows conclusions on the performance of both groups at follow-up. While the baseline findings were comparable between group A and B, several significant differences were found at follow-up. Accordingly, it can be presumed that the RCS teaching events would lead to increased self-perceived skills and the knowledge of undergraduate dental students after 3 months follow-up.

Additionally, it was not evaluated whether the primary positive outcome was achieved caused by the concept of RCS, the patient-case-based seminar, or the combination of both. The short observational period of 3 months does not allow conclusions on potential long-term effects. Furthermore, the self-reflection might have been more comprehensive and during the whole observational period to gain more insight into the students´ perspective and expectations. The current study focused on the risk-oriented prevention without addressing surgical issues, what is just one sub-aspect of the risk assessment and management in dental education and care. As a promising future approach, longitudinal curricular training in an interdisciplinary setting between dental disciplines and with interprofessional linking between dental and general medical students should be considered. Thereby, an implementation of the concept into medical education appears reasonable, too. Altogether, the results of this observational study can serve as a basis for the improvement of competence in the dental care of risk patients, as demanded in the literature [15, 31].

## Conclusion

This concept of learning risk-oriented prevention, including a specific RCS and its transfer into the patient treatment course, increased the self-perceived skills and the knowledge of undergraduate dental students after 3 months follow-up. These findings can serve as a basis for a further longitudinal curricular implementation within interprofessional teaching (dentistry and medicine), beginning in the early years of studies.

## Supplementary information

**Additional file 1 Supplementary Table 1.** Basis of applied risk classification system (mod. after Schmalz and Ziebolz 2020).

## Data Availability

The datasets used and/or analysed during the current study available from the corresponding author on reasonable request.

## Abbreviation

RCS: Risk classification system
