# Supplementary material for: Evaluation of an educational concept for risk-oriented prevention in undergraduate dental education
Source: BMC Med Educ. 2020 Sep 11;20:298. doi: 10.1186/s12909-020-02218-x (PMC7488547; doi:10.1186/s12909-020-02218-x)
Supplement: Supplementary file 1 — Additional file 1 Supplementary Table 1. Basis of applied risk classification system (mod. after Schmalz and Ziebolz 2020). [file 12909_2020_2218_MOESM1_ESM.docx]

**Supplementary table 1**: Basis of applied risk classification system (mod. after Schmalz and Ziebolz 2020).

| **Class** | | **Systemic diseases, medications, lifestyle factors** | |
| --- | --- | --- | --- |
|  |  | **Risk of complications** | **Risk of oral diseases** |
| **Low** | Definition | No increased risk of complications | No increased risk of oral disease |
|  | Examples | Generally healthy patient, no medication, no harmful lifestyle factors | |
| **Moderate** | Definition | Moderately increased risk of complications | Moderately increased risk of oral diseases |
|  | Examples | Well controlled COPD, oral bisphosphonate intake, alcohol consumption | Well controlled Diabetes mellitus (HbA1c <7%), Antihypertensive medication (risk of xerostomia), smoking <10 cigarettes/day |
| **High** | Definition | High risk of complications, potentially life-threatening risk | High risk of oral diseases |
|  | Examples | Heart valve replacement, immunosuppressive medication, drug consumption | Insufficiently controlled diabetes mellitus (HbA1c >7%), Cyclosporine A medication, smoking >10 cigarettes/day |
